# Primary cellular schwannoma of the kidney

**DOI:** 10.1111/pin.13370

**Published:** 2023-08-28

**Authors:** Xiaojing An, Jiong Liu, Bin Yang, Wei Zhang, Xiaoxia Dong

**Affiliations:** ^1^ Department of Pathology, Xiyuan Hospital China Academy of Chinese Medical Sciences Beijing China

**Abbreviations**
Bcl2: B cell lymphoma2
CDK4: cyclin dependent kinase 4
CS: cellular schwannoma
EMA: endomysial antibody
GFAP: glial fibrillary acidic protein
MDM2: mouse doubleminute 2 homolog
PGP9.5: protein gene product 9.5
SMA: sma monoclonal antibody
SRCC: sarcomatoid renal cell carcinoma
SS: synovial sarcoma
WI: weithted imaging

To the Editor,

Cellular schwannoma (CS), first reported by Woodruff in 1981,^1^ is an uncommon tumor derived from the Schwann cells of the peripheral nerves. Anatomically, schwannomas have a wide distribution, but the most frequent locations include the subcutaneous tissues of the extremities, head and neck regions, and retroperitoneal and mediastinal soft tissues. Primary CS of the kidneys is rare, and there are no typical clinical symptoms or imaging features reported. Therefore, CS is often misdiagnosed as a malignant tumor, resulting in overtreatment.

A 62‐year‐old woman was incidentally found to have a large mass in her right kidney during a medical examination for a health checkup. The patient had slight flank and colicky pain but no history of gross hematuria or related illnesses. Computed tomography images revealed a solid lesion protruding into the renal sinus in the middle of her left kidney. Magnetic resonance imaging showed an oval‐shaped abnormal shadow with a clear margin. The internal signal was uneven, with an equal T1 signal and a heterogeneous T2 signal. Diffusion‐weighted imaging (WI) showed an uneven high signal, a low‐signal capsule, and separation on T2WI (Figure 1a). A small low‐signal shadow was observed in the reverse phase, and the left renal pelvis was compressed and deformed. Multiple round cystic signals with clear boundaries were observed in the left kidney. The patient was suspected of having renal cell carcinoma and underwent radical nephrectomy.

**Figure 1:**
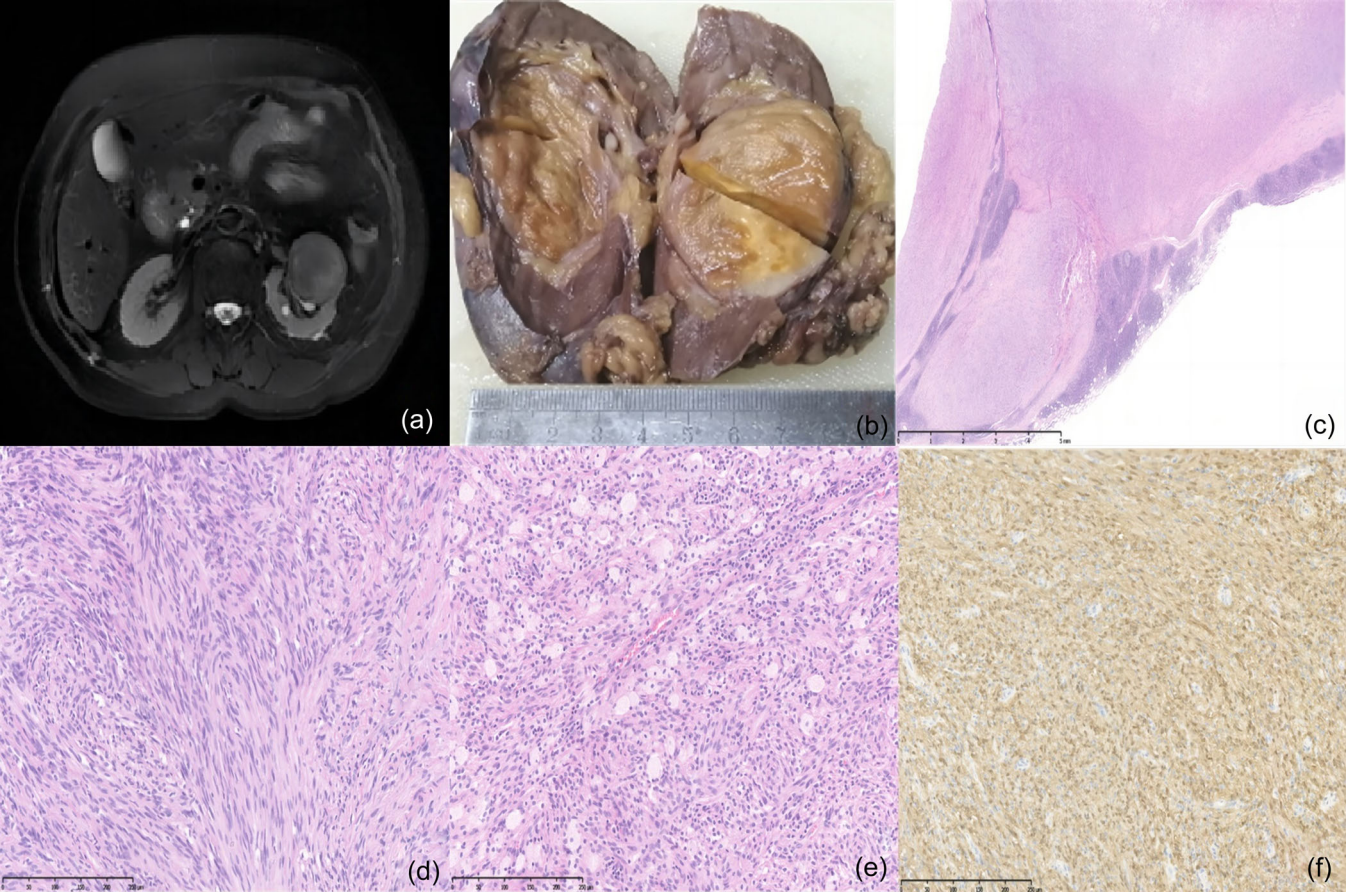
(a) A computed tomography image showing a solid nodule in the left kidney. (b) Grossly, the sectioned tumor appears yellow to brown with a clear margin. (c) The tumor appears multinodular and has a fibrous capsule with a lymphatic sheath outside the capsule. (d) The spindle tumor cells are arranged in a strip structure. (e) Foam cells and lymphocytes are found in the thick stromal. (f) The tumor cells exhibit immunopositivity for S100.

Surgical operation revealed a 5 × 3.5 × 2.6 cm mass located in the middle‐upper pole of the left kidney, which extended into the renal hilum. The mass was surrounded by a whitish fibrous capsule and had a grayish‐yellow, soft‐cut surface (Figure 1b). Microscopically, the mass was multinodular and surrounded by a discontinuous cuff of benign lymphoid hyperplasia and fibrous capsule. The tumor cells were arranged in short fascicules and had short‐spindle nuclei with slight pleomorphism. Mitotic figures were rare (1/10 high‐power fields), and nuclear atypia was absent. Many foamy histiocytes, scattered lymphocytes, and small amounts of thick‐walled blood vessels were also observed (Figure 1c–e). The differential diagnosis based on the histologic characteristics included synovial sarcoma (SS), smooth muscle tumor, solitary fibrous tumor, and malignant peripheral nerve sheath tumor.

Immunohistochemistry was performed: S100 (polyclonal; ZSGB‐BIO), SOX10 (clone EP268), GFAP (clone EP13), PGP9.5 (clone ZA‐0263), CD99 (clone EP8), Bcl‐2 (clone EP36), Vimentin (clone EP21), CK (clone AE1/AE3), HMB45 (clone HMB45), MDM2 (clone 1E6 and 17B3), Desmin (clone OTI4A8), CDK4 (clone EP180), CD34 (clone EP88), SMA (clone UMAB237), CD117 (clone EP10), EMA (clone GP14), Ki67 (clone UMAB107), and H3K27me3 (clone RM175).

The tumor cells were positive for S100, SOX10, GFAP, PGP9.5 (Figure 1f), CD99, Bcl‐2, and Vimentin. CD117 was expressed on mast cells. All other immunohistochemical results were negative (CK, HMB45, MDM2, Desmin, CDK4, CD34, SMA, CD117, and EMA), and nuclear expression of H3K27me3 was retained. The Ki67 nuclear proliferation index was approximately 20%. The SS18 (18q11) break was not detected by fluorescence in situ hybridization, and no specific tumor mutation was detected. The diagnosis of CS was based on histomorphological criteria, immunohistochemical data, and molecular genetics. At 24 months postoperatively, the patient was disease free.

Cellular schwannoma accounts for approximately 5% of schwannomas and is rarely involved with internal organs. In the English‐language literature, only six CS cases, involving patients aged 18 to 84 years, are reported.^2^, ^3^, ^4^ Most patients were asymptomatic, but a few had flank pain. The mass was mainly located in the central parenchyma of the kidney. The main nerves of the kidney consist of sympathetic and parasympathetic fibers that accompany the renal artery as it enters the renal hilum. This may explain why schwannomas are more frequently located in the renal hilum.

The histomorphological characteristics of CS are as follows. First, the tumor exhibits a well‐defined boundary and is surrounded by a fibrous envelope with lymphocytes forming a lymphatic sheath along this envelope. Second, the tumor cells show relatively uniform characteristics, forming a compact bundled structure without distinct Antoni A and B regions, Verocay bodies, or a palisade arrangement. Pseudosarcomatous cells with thickened and hyperchromatic nuclear chromatin are also observed. Furthermore, the mitotic index (1–4/high‐power field) is low. Third, hyalinization, foam cell aggregation, and lymphocyte infiltration into the thick stromal wall are present. The histopathological findings of this patient were consistent with most of the features reported in the literature. However, noteworthily, in this particular case, a multinodular growth pattern with three nodules visible was observed under the microscope. Additionally, a relatively monomorphic plump spindle cell proliferation was noted, arranged in short fascicule and without obvious thick‐walled vessels.

Cellular schwannomas are often characterized by their high concentration of tumor cells, displaying a spindle cell arrangement. Some of these cells may exhibit atypical features, such as single constructs, plump spindle cells, and the absence of thick‐wall vessels, which could lead to a misdiagnosis as SS. The diagnosis can be confirmed through a combination of characteristic morphology and immunohistochemical staining for S100, Sox10, and CD99. Most SS cells are positive for BCL‐2, CD99, CK, SS18‐SSX, and CD56 and focally positive for S100. More than 95% of SSs have the *SS18* (18q11) (*SYT*) gene breaks. Furthermore, it is important to exclude sarcomatoid renal cell carcinoma (SRCC) before diagnosing a spindle cell tumor of the kidney as CS. SRCC is a distinct subtype of renal cell carcinoma with higher malignancy, earlier invasion, and a higher tendency for metastasis than ordinary renal cell carcinoma and has a poor prognosis. Microscopically, the tumor tissue is composed of sarcomatoid and epithelioid components. The expression rate of broad‐spectrum cytokeratins in the sarcomatoid components of SRCC was 94%, and SRCC is an epithelial tumor with dual differentiation. In the present case, the tumor consisted of spindle cells and foam‐like histocytes with clear cytoplasm, which might be confused with clear cell carcinoma. CD68 could be combined with CK to distinguish SRCC from CS. Other spindle cell tumors of the kidney, such as myopericytoma, glomus tumor, angiomyolipoma, hemangiopericytoma, and leiomyosarcoma, were also excluded before making a final diagnosis based on the negative expression of HMB45, MDM2, Desmin, CD34, SMA, CD117, and EMA. Because of its benign clinical course, partial nephrectomy is a beneficial option if CS is diagnosed before radical surgery.

In summary, we reported a rare case of CS in the kidney with atypical histopathological features, which is similar to SS. Immunohistochemical staining or fluorescence in situ hybridization can help distinguish between the two. Given the typically benign clinical course of CS, it is advantageous to consider partial nephrectomy as a treatment option if the diagnosis can be made prior to radical surgery.

## AUTHOR CONTRIBUTIONS

Each author has participated in the work for the portions of the content during the course of this work. *Conception and writing of this manuscript*: Jiong Liu and Xiaojing An. Collection of clinical data: Wei Zhang and Xiaoxia Dong. Molecular analyses: Bin Yang. All authors read and approved the final version of the manuscript prior to submission.

## CONFLICT OF INTEREST STATEMENT

The authors declare no conflict of interest.

## ETHICS STATEMENT

This study was conducted in accordance with the Declaration of Helsinki of 1975. Written informed consent for research was obtained from the patient. Approval of the institutional ethics committee was not required for a case report, according to our institutional guidelines.
